# Prevalence and Characteristics of Multidrug-Resistant Livestock-Associated Methicillin-Resistant *Staphylococcus aureus* (LA-MRSA) CC398 Isolated from Quails (*Coturnix Coturnix Japonica*) Slaughtered for Human Consumption

**DOI:** 10.3390/ani11072038

**Published:** 2021-07-08

**Authors:** Vanessa Silva, Madalena Vieira-Pinto, Cândido Saraiva, Vera Manageiro, Lígia Reis, Eugénia Ferreira, Manuela Caniça, José L. Capelo, Gilberto Igrejas, Patrícia Poeta

**Affiliations:** 1 Department of Veterinary Sciences, University of Trás-os-Montes and Alto Douro (UTAD), 5000-801 Vila Real, Portugal; vanessasilva@utad.pt (V.S.); mmvpinto@utad.pt (M.V.-P.); candido.ls95@gmail.com (C.S.); 2 Department of Genetics and Biotechnology, Functional Genomics and Proteomics’ Unit, University of Trás-os-Montes and Alto Douro, 5000-801 Vila Real, Portugal; gigrejas@utad.pt; 3 Functional Genomics and Proteomics Unit, University of Trás-os-Montes and Alto Douro (UTAD), 5000-801 Vila Real, Portugal; 4 Associated Laboratory for Green Chemistry (LAQV-REQUIMTE), University NOVA of Lisboa, 2825-466 Caparica, Portugal; 5 Veterinary and Animal Research Centre, Associate Laboratory for Animal and Veterinary Science (AL4AnimalS), University of Trás-os-Montes and Alto Douro (UTAD), 5000-801 Vila Real, Portugal; 6 National Reference Laboratory of Antibiotic Resistances and Healthcare Associated Infections (NRL-AMR/HAI), Department of Infectious Diseases, National Institute of Health Dr. Ricardo Jorge, Av. Padre Cruz, 1649-016 Lisbon, Portugal; vera.manageiro@insa.min-saude.pt (V.M.); ligia.reis@insa.min-saude.pt (L.R.); Eugenia.Ferreira@insa.min-saude.pt (E.F.); manuela.canica@insa.min-saude.pt (M.C.); 7 Centre for the Studies of Animal Science, Institute of Agrarian and Agri-Food Sciences and Technologies, Oporto University, 4051-401 Oporto, Portugal; 8 BIOSCOPE Group, LAQV@REQUIMTE, Chemistry Department, Faculty of Science and Technology, NOVA University of Lisbon, 2825-466 Almada, Portugal; jlcm@fct.unl.pt; 9 Proteomass Scientific Society, 2825-466 Costa de Caparica, Portugal

**Keywords:** LA-MRSA, *Staphylococcus aureus*, CC398, poultry, quails

## Abstract

**Simple Summary:**

Methicillin-resistant *Staphylococcus aureus* (MRSA) is an important pathogen in both humans and animals worldwide. MRSA associated with livestock is a zoonotic pathogen that has been reported in several animals and, although its infections in humans are rare, this strain is recognized as an occupational hazard for people working in direct contact with livestock. Thus, we aimed to isolate MRSA from quails and to characterize their antimicrobial resistance and genetic lineages. One hundred swab samples were recovered from quails at the slaughterhouse. To investigate the prevalence and antimicrobial resistance of MRSA in poultry, we conducted this study on 100 quails slaughtered for human consumption. The antimicrobial resistance was investigated in all isolates as well as virulence genes and genetic lineages. Twenty-nine MRSA were isolated. The results showed that all MRSA isolates had resistance to multiple antibiotics. All strains were classified as livestock-associated. Most strains belonged to a well-known livestock-associated lineage: CC398.

**Abstract:**

Livestock-associated MRSA (LA-MRSA) is a zoonotic pathogen that has been reported in several animals, and it is often associated with clonal complex (CC) 398. We aimed to isolate MRSA from quails and to characterize their antimicrobial resistance and genetic lineages. One hundred swab samples were recovered from quails at the slaughterhouse. The swabs were inoculated onto CHROMagar™ MRSA agar plates for MRSA isolation. The presence of antimicrobial-resistant genes and virulence factors was investigated by PCR. All strains were typed by MLST, SCC*mec*-, *spa*- and *agr*-typing. From the 100 samples, 29 MRSA were isolated. All strains were resistant to penicillin, cefoxitin, ciprofloxacin, erythromycin and clindamycin and carried the *bla*Z, *mec*A, *erm*B and *erm*C genes. All strains, except one, showed resistance to tetracycline and harbored the *tet*M, *tet*K and *tet*L genes in different combinations. Twenty strains belonged to ST398 and SCC*mec* type V, and nine strains belonged to the new ST6831. Twenty-eight out of twenty-nine strains were ascribed to t011 and one to t108. As far as we know, this is the first report of MRSA from quails slaughtered for human consumption. Most strains belonged to ST398-t011, which is the most common LA-MRSA clone found in livestock in Europe.

## 1. Introduction

*Staphylococcus aureus* is a commensal organism that is widely disseminated among humans and other mammals. However, *S. aureus* can also be an opportunistic pathogen that is responsible for a wide range of infections, including skin and soft tissue infections, osteomyelitis, endocarditis and sepsis [1]. Besides, this pathogen has the ability to easily acquire antimicrobial resistance determinants, and it is often associated with several virulence factors [2]. The acquisition of staphylococcal cassette chromosome *mec* (SCC*mec*) elements containing the *mec* genes is responsible for the development of other *S. aureus* strains, namely the methicillin-resistant *S. aureus* (MRSA) [3]. MRSA strains are also resistant to all ß-lactam agents, including cephalosporins and carbapenems, and are often associated with resistance to other classes of antimicrobial agents [4,5]. S. *aureus* is widely disseminated among humans and the environment, and it is known that it can spread through the air, water, food, contaminated surfaces and direct contact between humans and animals [6,7,8,9]. Furthermore, both methicillin-susceptible *S. aureus* (MSSA) and MRSA strains have been found colonizing and infecting pets and wild animals, including hares, rats, foxes, birds, and livestock such as pigs, cattle and poultry [2,10]. Studies have shown that MRSA isolated from animals were significantly more resistant to tetracycline, clindamycin, ciprofloxacin and gentamicin than strains isolated from humans [4,11]. Livestock-associated MRSA (LA-MRSA) poses a zoonotic risk for consumers and particularly for those working in close contact with livestock [12]. The use of multilocus sequence typing (MLST) allowed the tracing of evolutionary origin and spread of MRSA [3]. Among animal MRSA strains, the clonal lineage clonal complex (CC) 398 is considered the most notable and widespread LA-MRSA strain in Europe and North America [12]. In contrast, studies have shown that the most widespread LA-MRSA strain in Asia belongs to ST9 [13,14]. Other lineages have been reported in livestock, including CC97, CC133 and CC522 isolated mainly from ruminants and ST385 from poultry [3]. It is believed that *S. aureus* CC398 originated from humans. However, this strain was transmitted to livestock, and it acquired the *mec*A gene becoming MRSA [15]. Nevertheless, strains of CC398 MRSA are rarely associated with infections in livestock [3]. MRSA CC398 was first described in swine, but it has been isolated from pets, humans and other livestock animals such as cattle and poultry [16,17,18]. 

The European Union banned the use of antibiotics as growth promoters in 2006 due to the increase and spread of antimicrobial-resistant bacteria [19]. Nevertheless, nowadays, antibiotics are still prescribed by veterinarians to treat illnesses. In 2015, a total of 8361 tons of antimicrobial agents were used in veterinary practices in the EU [20]. Tetracycline, followed by penicillin, was the most prescribed antibiotics for food-producing animals, according to the ECDC/EFSA/EMA report in 2017 [21]. Although the use of antibiotics in poultry farming is controversial due to their impact on public health, a sustainable poultry industry is not possible without the use of antimicrobials [22,23]. Commercial quail (*Coturnix coturnix japonica*) is the smallest poultry species farmed for human consumption. Quail meat is very appreciated by consumers due to its taste and also due to its low-fat content and good levels of phospholipids [24]. However, studies on the prevalence of antimicrobial-resistant bacteria in quails and quail meat are still very scarce. In Portugal, MRSA and MSSA CC398 were previously reported in pigs, calves, humans and wild rodents [25,26,27,28]. Nevertheless, the prevalence of MRSA has not yet been studied in poultry in Portugal. Therefore, in this study, we investigated the prevalence of MRSA in quails at the slaughterhouse level and aimed to characterize the antimicrobial resistance, virulence and genetic lineages of the isolates.

## 2. Materials and Methods

### 2.1. Sample Collection and Bacterial Isolates

In February 2020, a total of 100 samples were collected from quails in a Portuguese slaughterhouse. Samples were collected from Cloaca and trachea using only one swab per animal. Batches of quails arrived at the slaughterhouse 3 days a week, and around 36,000 animals were slaughtered each day. Each batch carried around 15,000 quails. Four samples were recovered from each batch. The swabs were inoculated into Brain Heart Infusion (BHI) broth with 6.5% of NaCl and incubated at 37 °C under aerobic conditions and examined after 24 h. The inoculum was then seeded onto CHROMagar™ MRSA agar plates and incubated at 37 °C for 24 to 48 h. Three colonies per plate with specific color and morphology were recovered and further investigated. The species confirmation was performed first by biochemical tests and then by MALDI-TOF (Bruker Daltonics, Bremen, Germany).

### 2.2. Antimicrobial Susceptibility Testing

The antimicrobial susceptibility testing was performed by the Kirby Bauer disk diffusion method, which followed the recommendations given in the European Committee on Antimicrobial Susceptibility Testing (EUCAST) 2019 guidelines with the exception of kanamycin that followed the Clinical and Laboratory Standards Institute (CLSI) 2017 standards. The following antibiotic discs were used: cefoxitin (30 μg), chloramphenicol (30 μg), ciprofloxacin (5 μg), clindamycin (2 μg), erythromycin (15 μg), fusidic acid (10 μg), gentamicin (10 μg), kanamycin (30 μg), linezolid (10 μg), mupirocin (200 μg), penicillin (1U), tetracycline (30 μg), tobramycin (10 μg) and trimethoprim/sulfamethoxazole (1.25/23.75 μg). The reference strain *S. aureus* ATCC^®^ 25923 was used as a quality control strain.

### 2.3. Antimicrobial Resistance and Virulence Genes

Prior to DNA extraction, isolates were grown on BHI agar and incubated at 37 °C for 18 h. Bacterial cells were enzymatically lysed, and DNA extraction was performed as previously described [29]. The extracted DNA was stored in a freezer at −20 °C until used. Methicillin resistance was confirmed by PCR with primers targeting the *mec*A gene as previously described [30]. All isolates were evaluated for the presence of antimicrobial-resistant genes, as previously described (Table S1), that encode resistance to penicillin (*bla*Z), tetracyclines (*tet*M, *tet*L, *tet*K and *tet*O), aminoglycosides (*aac*(6′)-Ie-*aph*(2′’)-Ia, *aph*(3′)-IIIa, *ant*(4′)-Ia and *str*), macrolides and lincosamides (*erm*A, *erm*B, *erm*C, *erm*T, *mph*C, *msr*(A/B), *lnu*A, *lnu*B, *vga*A and *vga*B), fusidic acid (*fus*A, *fus*B and *fus*C) and chloramphenicol (*fex*A, *fex*B, *catp*C194, *catp*C221 and *catp*C223).

The presence of the virulence genes encoding Panton–Valentine leucocidin (PVL) (*lukF*/*lukS*-PV), alpha-, beta- and delta-hemolysins (*hla*, *hlb* and *hld*), exfoliative toxins (*eta* and *etb*) and toxic shock syndrome toxin (*tst*) was also studied by PCR (Table S1). The *scn* gene is a marker of the immune evasion cluster (IEC) system since it is common to all IEC groups, and its presence was studied in all isolates. After a positive result, the presence of the *chp*, *sak*, *sea* and *sep* genes was studied to determine the IEC group [31].

Positive and negative controls used in all experiments belonged to the strain collection of the University of Trás-os-Montes and Alto Douro.

### 2.4. Molecular Typing

All isolates were typed by *spa* typing using specific primers and conditions as previously described [32]. The sequences were analyzed using the BioNumerics^©^ Applied Maths software, and *spa* types were identified using the database available at http://spatyper.fortinbras.us (accessed on 20 May 2021). MLST was performed in all isolates, and it was based on seven housekeeping genes (*arc*C, *aro*E, *glp*F, *gm*K, *pta*, *tpi*A and *yqi*L) as described in the MLST database and by Enright et al. [33]. Isolates were assigned to a sequence type (ST) and a clonal complex (CC) according to the MLST database (https://pubmlst.org accessed on 24 May 2021). The *agr* type of all isolates was determined by the PCR as described by Shopsin et al. [34]. All isolates were characterized by SCC*mec* typing (I–V) using specific primers [35].

## 3. Results

### 3.1. Antimicrobial Resistance and Virulence

**Figure 1:**
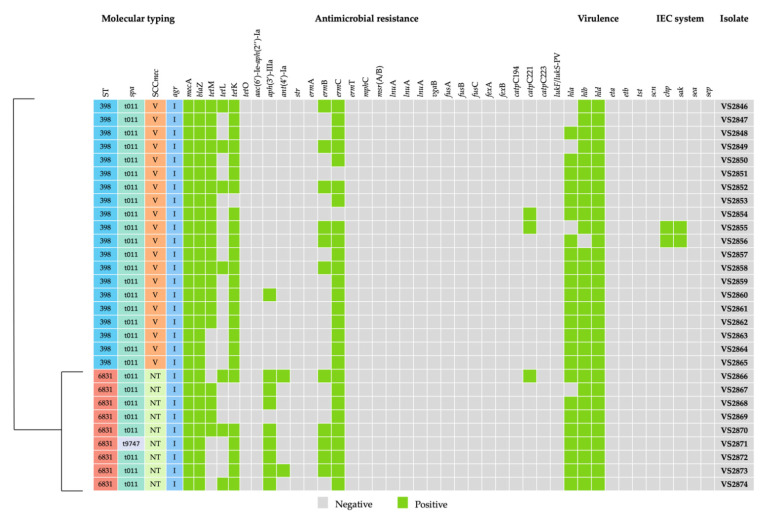
Genotypic profile of antibiotic resistance genes, virulence factors, and genetic diversity detected in each of the 29 LA-MRSA strains isolated from slaughtered for human consumption. ST: sequence type; N.T: Not typeable.

A total of 100 quail samples were used in this study. From the 100 samples, 29 were positive for MRSA. All strains harbored the *mec*A gene, which confers resistance to methicillin. Regarding the phenotypic resistance, eight different resistance phenotypes were detected. All isolates were considered multidrug-resistant since they were resistant to at least three classes of antimicrobial agents. Furthermore, all 29 MRSA isolates were resistant to penicillin, ciprofloxacin, erythromycin and clindamycin. All isolates carried the beta-lactam resistance gene *bla*Z. A summary of the carriage of resistance genes is provided in Figure 1. Twenty-seven isolates harbored macrolide–lincosamide resistant genes, such as the *erm*C (n = 17) or the combination of *erm*B and *erm*C genes (n = 10). No other macrolide or lincosamide resistance gene was detected in this study. Resistance to tetracycline was detected in 28 out of 29 MRSA isolates. The presence of four tetracycline resistance genes was investigated, and *tet*M, *tet*K and *tet*L were detected in different combinations. Four strains carried the *tet*M gene alone, whereas three strains carried the *tet*K, and another three carried the *tet*L. Ten out of twenty-eight tetracycline-resistant isolates carried both *tet*K and *tet*M genes, three isolates carried *tet*K and *tet*L, and five strains carried all three genes. Resistance to aminoglycosides was identified in nine isolates that harbored the *aph*(3′)-IIIa and *ant*(4′)-Ia genes. Three isolates showed resistance to chloramphenicol conferred by the *catp*C221 gene. Finally, five isolates were resistant to fusidic acid; however, none of the strains carried any of the genes tested. Regarding the presence of virulence factors, all strains were negative for the genes encoding for PVL (*lukF*/*S-PV*), toxic shock syndrome toxin and exfoliative toxins. All strains harbored at least two genes encoding for the hemolysins, including *hla* (n = 24), *hlb* (n = 28) and *hld* (n = 29). Only two strains (VS2855 and VS2856) carried the *scn* gene, which is the marker of the IEC system. The presence of the other IEC genes was further investigated in those strains. However, both isolates carried only the *sak* gene, and therefore it was not possible to assign the IEC type.

### 3.2. Molecular Typing

The most common lineage among quail isolates was ST398 (Figure 1). In fact, only two different STs were found in this study. Of the 29 isolates, 20 belonged to ST398, whereas the remaining 9 were ascribed to ST6831 which was first described in this study. Regarding the *spa*-typing, 28 out of 29 isolates belonged to t011, and 1 isolate, belonging to ST6831, was ascribed to t9747. All strains were typed as *agr* I. Isolates ascribed to ST398 harbored SCC*mec* type V elements, while isolates belonging to ST6831 were not typeable.

## 4. Discussion

In this study, we obtained a prevalence of MRSA of almost 30% in quails (*Coturnix coturnix japonica*) slaughtered for human consumption. As far as we know, no other study has reported the prevalence and carriage of MRSA in quails at the slaughterhouse level. Very few studies have been carried out so far in quails, and they report the presence of MRSA strains in quail eggshells or meat [36,37,38]. Saadati et al. (2019) sampled meat from 70 quails randomly collected from the shopping centers in Iran and found only two (2.86%) MRSA strains [38]. Poultry, and poultry meat in general, seem to frequently carry MRSA strains. A study conducted in the Netherlands detected MRSA in 11 (4.4%) out of the 250 pooled throat swabs of broilers [39]. Bounar-Kechih et al. (2018) analyzed the prevalence of MRSA in laying hens and broiler chickens samples taken at the slaughterhouse and reported the presence of MRSA in 23.9% and 6.4% of samples, respectively [40]. In a long-term study, conducted between 2011 and 2018, 4248 nasal swabs were recovered from breeding hens (n = 654), laying hens (n = 838), broilers (n = 1614) and turkeys (n = 1142), and MRSA was detected in 252 (5.9%) of those samples [41]. A study carried out in Denmark detected the presence of MRSA in 4% of 102 chicken meat samples [42]. A total of 61 chicken (n = 50) and turkey (n = 11) meat samples were obtained from retail stores in the North West England, and MRSA was recovered from nine (7.3%) samples [43]. Chilled retail chicken (n = 114) and turkey (n = 53) samples were used in a study conducted in the USA, and only two (1.2%) MRSA strains were detected [44]. There are no studies available in healthy quail that allow us to make a direct comparison of the prevalence of MRSA in these birds. The studies mentioned above, which were all conducted with poultry samples, reported a lower prevalence of MRSA (1.2% to 23.9%) compared to the one obtained in our study (30%). This result may be due to the fact that, in Portugal, the legislation for the administration of antibiotics in quails is not as well-regulated as for other poultry, such as broilers. Therefore, a higher amount of antibiotics may be administrated indiscriminately to these birds, resulting in the selection of antimicrobial-resistant strains. In fact, all MRSA isolates from this study had a multidrug-resistant profile. The high diversity of resistance is probably mainly due to the long-term usage of different antimicrobial classes in the agricultural sector [45,46]. Resistance to penicillin, ciprofloxacin, erythromycin and clindamycin was detected in all isolates and all isolates, except one, also showed resistance to tetracycline. These results are in accordance with the 2017 ECDC/EFSA/EMA report that stated that tetracycline and penicillin were the most prescribed antibiotics for livestock [21]. The high level of resistance to the quinolone investigated in the current study is in accordance with other studies about the poultry sector conducted in Europe [45,46]. *tet*M, *tet*K and *tet*L genes were detected in different combinations in tetracycline-resistant isolates. It has been shown that *tet*M and *tet*O genes are located in transposons or chromosomes, while *tet*K and *tet*L genes are located in plasmids [47]. In fact, several resistance genes are often assembled together on mobile genetic elements. Therefore, the selective pressure caused by just one antibiotic may drive the resistance to another antibiotic, which leads to co-resistance [48]. Some of the plasmids carrying tetracycline-resistant genes may carry additional genes. It has been shown that the same plasmids carrying the *tet*L gene may also carry the *dfrK* gene, which confers resistance to trimethoprim and to the macrolide–lincosamide resistant gene *erm*B [49]. In our study, we did not detect resistance to trimethoprim-sulfamethoxazole; however, all isolates (except one) that carried the *tet*L gene also carried the *erm*B gene. Nevertheless, the most frequent macrolide–lincosamide resistance gene was *erm*C. This gene is often located in small plasmids [49]. These antimicrobials are among the most frequently used in the poultry industry, and resistance to these antimicrobials is often detected in poultry isolates [50]. Although all isolates in our study showed macrolide–lincosamide resistance, there were two isolates that did not harbor any of the resistance genes tested. This result has been previously reported in strains from diseased pigs belonging to CC398 [51]. Resistance to penicillin was conferred by the *bla*Z gene, which was present in all isolates. Studies have shown that the administration of amoxicillin to poultry was associated with resistance to beta-lactams and other antimicrobials, such as aminoglycosides and chloramphenicol [52]. In the current study, nine isolates were resistant to aminoglycosides, but only one isolate had resistance to chloramphenicol simultaneously. Although chloramphenicol administration was banned in Europe in 1997, we found three isolates resistant to this antimicrobial, and all harbored the *catp*C221 gene. This finding might be explained by the fact that the use wide of broad-spectrum antibiotics may have exacerbated the co-selection of resistance genes [53,54]. LA-MRSA strains, such as those belonging to CC398, usually lack virulence genes that cause severe human infections, such as the IEC genes, the genes encoding the toxic shock syndrome toxin and the Panton–Valentine leucocidin (PVL) [55]. In fact, CC398 strains are associated with high levels of antimicrobial-resistant genes, often contrasting with the low detection of virulence genes [27,56]. Indeed, our isolates lacked both *tst* and PVL genes. However, two isolates, both belonging to CC398 and t011, were positive for the *scn* gene, which is the marker of the IEC system. Nevertheless, both isolates harbored only the *sak* gene, in addition to *scn*, and it was not possible to ascribe the IEC type. It has been shown that LA-MRSA CC398 emerged from humans, and it has jumped to livestock, losing a bacteriophage (ΦSa3) which harbors the *scn* gene [15,57]. Nevertheless, studies have reported the presence of the IEC system in MRSA CC398 isolated from animals, including pigs, poultry, horses and wild boars [16,57,58,59]. Our findings suggest that the IEC has been reacquired, as described by others [57,58]. Nevertheless, both IEC-positive isolates carried the *tet* genes, particularly *tet*M and *tet*K, which are considered the hallmark of the livestock CC398 clade [15]. Twenty isolates belonged to ST398, *spa*-type t011, and carried the SCC*mec* type V cassette. MRSA ST398 has been spreading through Europe since 2005, and it is often associated with specific *spa*-types, such as t011, t034, t108, t567, t899, t1197 and t2346 [16]. Strains belonging to ST398 and *spa*-type t011 are very common in livestock, including pigs, cattle and poultry, and have been reported in numerous studies in Europe. To our knowledge, none of the studies conducted in MRSA from quails analyzed the ST and *spa*-types of the strains. However, several studies conducted in poultry and poultry products showed that ST398-t011-SCCmec *V* is the major lineage in poultry [60,61,62,63]. Other STs, such as those belonging to CC5, commonly found among poultry were not detected in this study [63]. Still, 9 of the 29 MRSA isolated in our study were ascribed to the new ST6831. One of those isolates belonged to *spa*-type t9747 and is a one-locus variant of *spa*-type t108 commonly associated with ST398. As far as we know, t9747 was reported only once in 2013, but no MLST data is available [64]. Finally, all isolates belonging to ST398 or the new ST6831 were ascribed to *agr* type I. In contrast, Kraushaar et al. (2017) reported that all CC398 isolates from poultry belonged to agr II [58]. Most studies conducted in poultry and poultry meat do not report the agr type [43,61,62]. Nevertheless, CC398 seems to be associated with *agr* I in isolates from other livestock [27,65,66].

## 5. Conclusions

A moderate frequency of MRSA (29%) was found among quails slaughtered for human consumption. All strains were multidrug-resistant and had a remarkable diversity of antimicrobial resistance and resistant genes. Nearly all isolates that showed resistance to tetracycline had harbored a diversity of *tet* genes in different combinations, which is a marker of LA-MRSA CC398 strains. The indiscriminative use of antimicrobials in quail production, particularly those considered to be essential in human medicine, may be favorable to the sector, but it will likely contribute to the increase and spread of antimicrobial-resistant pathogens. Therefore, more restrictive legislation should be implemented in all poultry sectors. Furthermore, frequent monitoring of MRSA strains from poultry and other livestock is essential to understand the spread and the changes of the genetic repertoire, as well as the zoonotic potential of these strains.

## Supplementary Materials

The following are available online at https://www.mdpi.com/article/10.3390/ani11072038/s1, Table S1: Primer pairs used for molecular typing and detection of antimicrobial-resistant genes in MRSA strains.
