# Supplementary material for: Prevalence and Characteristics of Multidrug-Resistant Livestock-Associated Methicillin-Resistant Staphylococcus aureus (LA-MRSA) CC398 Isolated from Quails (Coturnix Coturnix Japonica) Slaughtered for Human Consumption
Source: Animals (Basel). 2021 Jul 8;11(7):2038. doi: 10.3390/ani11072038 (PMC8300319; doi:10.3390/ani11072038)
Supplement: Supplementary file 1 [file animals-11-02038-s001.zip › animals-1288435-supplementary.pdf]

Table S1. Primer pairs used for molecular typing and detection of antimicrobial resistance genes in MRSA strains.

Target gene	Primer sequence (5'-3')	Amplicon size (bp)	Reference
**Antimicrobial resistance**			
mecA	F: GGGATCATAGCGTCATTATTC<br>R: AACGATTGTGACACGATAGCC<br>R: AGACCCGGGAACGTATTCAC	527	[1]
blaZ	F: CAGTTCACATGCCAAAGAG<br>R: TACACTCTTGGCGGTTTC	772	[2]
***erm*(A)**	F: TCTAAAAAGCATGTAAAAGAA<br>R: CTTCGATAGTTTATTAATATTAG	645	[3]
***erm*(B)**	F: GAAAAGTACTCAACCAAATA<br>R: AGTAACGGTACTTAAATTGTTTA	639	[4]
***erm*(C)**	F: TCAAAACATAATATAGATAAA<br>R: GCTAATATTGTTTAAATCGTCAAT	642	[3]
***erm*(T)**	F: CCGCCATTGAAATAGATCCT<br>R: TTCTGTAGCTGTGCTTTCAAAAA	200	[5]
***msr*(A/B)**	F: GCAAATGGTGTAGGTAAGACAACT<br>R: ATCATGTGATGTAAACAAAAT	399	[6]
***mph*(C)**	F: ATGACTCGACATAATGAAAT<br>R: CTACTCTTTCATACCTAACTC	900	[2]

***lnu*A**	F: GGTGGCTGGGGGGTAGATGTATTAACTGG<br>R: GCTTCTTTTGAAATACATGGTATTTTTCGATC	323	[7]
***lnu*B**	F: CCTACCTATTGTTTGTGGAA<br>R: ATAACGTTACTCTCCTATTC	944	[8]
***vga*A**	F: AGTGGTGGTGAAGTAACACG<br>R: GGTTCAATACTCAATCGACTGAG	1264	[9]
***vga*B**	TGACAATATGAGTGGTGGTG<br>GCGACCATGAAATTGCTCTC	576	[10]
***tet*(K)**	F: TTAGGTGAAGGGTTAGGTCC<br>R: GCAAACTCATTCCAGAAGCA	697	[11]
***tet*(L)**	F: CATTTGGTCTTATTGGATCG<br>R: ATTACACTTCCGATTTCGG	456	[11]
***tet*(M)**	F: GTTAAATAGTGTTCTTGGAG<br>R: CTAAGATATGGCTCTAACAA	576	[11]
***tet*(O)**	F: GATGGCATACAGGCACAGAC<br>R: CAATATCACCAGAGCAGGCT	615	[11]
aac(6´)-Ie-aph(2´´)-Ia	F: CCAAGAGCAATAAGGGCATA<br>R: CACTATCATAACCACTACCG	220	[12]
aph(3´)-IIIa	F: GCCGATGTGGATTGCGAAAA<br>R: GCTTGATCCCCAGTAAGTCA	292	[12]

ant(4)-Ia	F: GCAAGGACCGACAACATTTC<br>R: TGGCACAGATGGTCATAACC	165	[12]
str	F: TATTGCTCTCGAGGGTTC<br>R: CTTTCTATATCCATTCATCTC	646	[13]
***fex*A**	F: GTACTTGTAGGTGCAATTACGGCTGA<br>R: CGCATCTGAGTAGGACATAGCGTC	1272	[14]
fexB	F: TTCCCACTATTGGTGAAAGGAT<br>R: GCAATTCCCTTTTATGGACGTT	816	[15]
***catp*C194**	F: CGACTTTTAGTATAACCACAGA<br>R: GCCAGTCATTAGGCCTAT	570	[13]
***catp*C221**	F: ATTTATGCAATTATGGAAGTTG<br>R: TGAAGCATGGTAACCATCAC	434	[13]
***catp*C223**	F: GAATCAAATGCTAGTTTTAACTC<br>R: ACATGGTAACCATCACATAC	283	[13]
**Virulence**			
hla	F: CTGATTACTATCCAAGAAATTCGATTG<br>R: CTTTCCAGCCTACTTTTTTATCAGT	209	[16]
hlb	F: GTGCACTTACTGACAATAGTGC<br>R: GTTGATGAGTAGCTACCTTCAGT	309	[16]
hld	F: AAGAATTTTTATCTTAATTAAGGAAGGAGTG	456	[16]

	R: TTAGTGAATTTGTTCACTGTGTCGA		
eta	F: ACTGTAGGAGCTAGTGCATTTGT<br>R: TGGATACTTTTGTCTATCTTTTTCATCAAC	190	[16]
etb	F: CAGATAAAGAGCTTTATACACACATTAC<br>R: AGTGAACTTATCTTTCTATTGAAAAACACTC	612	[16]
tst	F: TTCACTATTTGTAAAAGTGTCAGACCCACT<br>R: TACTAATGAATTTTTTTATCGTAAGCCCTT	180	[17]

[1] Zhang, K., Sparling, J., Chow, B.L., Elsayed, S., Hussain, Z., Church, D.L., Gregson, D.B., Louie, T., Conly, J.M., 2004, New quadriplex PCR assay for detection of methicillin and mupirocin resistance and simultaneous discrimination of Staphylococcus aureus from coagulase-negative staphylococci. J Clin Microbiol 42, 4947-4955.

[2] Schnellmann, C., Gerber, V., Rossano, A., Jaquier, V., Panchaud, Y., Doherr, M.G., Thomann, A., Straub, R., Perreten, V., 2006, Presence of new mecA and mph(C) variants conferring antibiotic resistance in Staphylococcus spp. isolated from the skin of horses before and after clinic admission. J Clin Microbiol 44, 4444-4454.

[3] Sutcliffe, J., Grebe, T., Tait-Kamradt, A., Wondrack, L., 1996, Detection of erythromycin-resistant determinants by PCR. Antimicrob Agents Chemother 40, 2562-2566.

[4] Shopsin, B., Mathema, B., Alcabes, P., Said-Salim, B., Lina, G., Matsuka, A., Martinez, J., Kreiswirth, B.N., 2003, Prevalence of agr specificity groups among Staphylococcus aureus strains colonizing children and their guardians. J Clin Microbiol 41, 456-459.

[5] Gomez-Sanz, E., Torres, C., Lozano, C., Fernandez-Perez, R., Aspiroz, C., Ruiz-Larrea, F., Zarazaga, M., 2010, Detection, molecular characterization, and clonal diversity of methicillin-resistant Staphylococcus aureus CC398 and CC97 in Spanish slaughter pigs of different age groups. Foodborne Pathog Dis 7, 1269-1277.

[6] Wondrack, L., Massa, M., Yang, B.V., Sutcliffe, J., 1996, Clinical strain of Staphylococcus aureus inactivates and causes efflux of macrolides. Antimicrob Agents Chemother 40, 992-998.

[7] Lina, G., Quaglia, A., Reverdy, M.E., Leclercq, R., Vandenesch, F., Etienne, J., 1999. Distribution of genes encoding resistance to macrolides, lincosamides, and streptogramins among staphylococci. Antimicrob. Agents Chemother. 43, 1062–1066.

[8] Bozdogan, B., Berrezouga, L., Kou, M.S., Yurek, D.A., Farley, K.A., Stockman, B.J., Leclercq, R., 1999. A new resistance gene, linB, conferring resistance to lincosamides by nucleotidylation in Enterococcus faecium HM1025. Antimicrob. Agents Chemother. 43, 925–929.

[9] Lozano C, Aspiroz C, Rezusta A, Gómez-Sanz E, Simon C, Gómez P, Ortega C, Revillo MJ, Zarazaga M, Torres C. Identification of novel vga(A)-carrying plasmids and a Tn5406-like transposon in meticillin-resistant Staphylococcus aureus and Staphylococcus epidermidis of human and animal origin. Int J Antimicrob Agents. 2012 Oct;40(4):306-12.

[10] Hammerum, A.M., Jensen, L.B., Aarestrup, F.M., 1998. Detection of the satA gene and transferability of virginiamycin resistance in Enterococcus faecium from food-animals. FEMS Microbiol. Lett. 168, 145–151.

[11] Aarestrup, F.M., Agers, L.Y., Ahrens, P., JC, J.L., Madsen, M., Jensen, L.B., 2000, Antimicrobial susceptibility and presence of resistance genes in staphylococci from poultry. Vet Microbiol 74, 353-364.

[12] Van de Klundert, J.A.M., Vliegenthart, J.S., 1993. PCR detection of genes coding for aminoglycoside-modifying enzymes, in: Persing, D.H., Smith, T.F., Tenover, F.C., White, T.J. (Eds.), Diagnostic Molecular Microbiology: Principles and Applications. American Society for Microbiology, Washington D.C., pp. 547–552.

[13] Schnellmann, C., Gerber, V., Rossano, A., Jaquier, V., Panchaud, Y., Doherr, M.G., Thomann, A., Straub, R., Perreten, V., 2006. Presence of new mecA and mph(C) variants conferring antibiotic resistance in Staphylococcus spp. isolated from the skin of horses before and after clinic admission. J. Clin. Microbiol. 44, 4444–4454.

[14] Kehrenberg, C., Schwarz, S., 2006. Distribution of Florfenicol Resistance Genes fexA and cfr among Chloramphenicol-Resistant Staphylococcus Isolates. Antimicrob. Agents Chemother. 50, 1156–1163.

[15] Liu, H., Wang, Y., Wu, C., Schwarz, S., Shen, Z., Jeon, B., Ding, S., Zhang, Q., Shen, J., 2012. A novel phenicol exporter gene, fexB, found in enterococci of animal origin. J. Antimicrob. Chemother. 67, 322–325.

[16] Jarraud, S., Mougel, C., Thioulouse, J., Lina, G., Meugnier, H., Forey, F., Nesme, X., Etienne, J., Vandenesch, F., 2002. Relationships between Staphylococcus aureus genetic background, virulence factors, agr groups (alleles), and human disease. Infect. Immun. 70, 631–641.

[17] Yamaguchi, T., Nishifuji, K., Sasaki, M., Fudaba, Y., Aepfelbacher, M., Takata, T., Ohara, M., Komatsuzawa, H., Amagai, M., Sugai, M., 2002. Identification of the Staphylococcus aureus etd pathogenicity island which encodes a novel exfoliative toxin, ETD, and EDIN-B. Infect. Immun. 70, 5835–5845.
